# Beneath the surface: DNA barcoding of shark fins in Singapore

**DOI:** 10.1098/rsos.240532

**Published:** 2024-09-04

**Authors:** Manisha Saigal, Hannah Ng Shueh Yi, Nageen Ayesha Rameez, Siebe van Manen, Bui Tr Van Anh, Vidhi P. Arora, Kai Ding Max Han, Jenelle Qian Tong Lee, Adlan Syaddad, Claudia Kexin Tan, Elisa X. Y. Lim, Benjamin J. Wainwright

**Affiliations:** ^1^ Yale-NUS College, National University of Singapore, Singapore; ^2^ University College Utrecht, Utrecht University, Utrecht, The Netherlands; ^3^ Fulbright University Vietnam, Ho Chi Minh City, Vietnam; ^4^ Department of Biological Sciences, National University of Singapore, Singapore

**Keywords:** elasmobranchii, shark fin, conservation, *Carcharhinus*, CITES, IUCN

## Abstract

The global decline of shark populations, largely driven by overfishing to supply the shark fin trade, poses a significant threat to marine ecosystems. Southeast Asia, and particularly Singapore, is a key hub for the transit and trade of shark fins that contribute to the exploitation of these apex predators. Through the use of DNA barcoding techniques, this study aimed to determine what species of shark are involved in the Singapore shark fin trade. Fins were collected from markets, dried goods shops and traditional Chinese medicine halls throughout Singapore. In total, DNA was extracted from 684 fins collected in January 2024 and PCR amplification targeted a fragment of the mitochondrial COI gene for species identification. Results revealed fins from 24 species across 16 genera, with 19 species listed on CITES Appendices II, and 16 listed as threatened on the IUCN Red List (critically endangered = 2, endangered = 4, vulnerable = 10). The top five most frequently identified species were *Carcharhinus falciformis*, *Galeorhinus galeus*, *Rhizoprionodon oligolinx, Sphyrna lewini* and *Rhizoprionodon acutus*. Of these, four are listed on CITES Appendix II and four are listed as threatened on the IUCN Red List.

## 1. Introduction

As a result of widespread exploitation, global shark populations have suffered declines of more than 70% since 1970 [1]. Compounding these declines, many shark species have life history characteristics (e.g. late maturity, low birth rates and prolonged gestation periods) that increase their susceptibility to fishing pressures and the risk of extinction [2–4]. The primary driver of the decline in shark populations is overfishing, mainly due to the unsustainable global trade in shark fins and increasingly meat. Consequently, two-thirds of the shark species traded on international markets are at risk of extinction [5–7].

Worth approx. $314 million, the global shark fin trade results in the death of an estimated 100 million sharks annually [8–10]. Shark fins are sourced from various locations across the globe and are then transported through regional and international trade routes with multiple connecting links and hubs [10–13], which makes them prone to mislabelling and illegal trade [14,15]. Accordingly, the Convention on International Trade in Endangered Species of Wild Fauna and Flora (CITES) attempts to address the complexities of these global supply chains through key multilateral environmental agreements involving 183 states and the European Union [16]. Through the use of three appendices that provide varying levels of protection based on the species' evaluated threat level, CITES aims to ensure that such trade is legal, sustainable and traceable [17]. In 2023, CITES implemented a decision to add 54 species from the requiem shark family (*Carcharhinidae* spp.) and six species from the hammerhead family (*Sphyrna* spp.) to its appendices. Importantly, this update covered sharks that all appear highly similar when attempting to use morphological characteristics in their identification; a visual similarity which has previously complicated species identification in these groups. However, these new listings should simplify and facilitate improved enforcement efforts and enable more accurate record-keeping.

Southeast Asia is a global production and trading hotspot where shark and ray biodiversity are especially threatened [18–22]. In particular, Singapore is a globally significant transit and trade hub for both legal and illegal shark products [18,19,23,24], and neighbouring countries such as Indonesia and Malaysia are recognized as having important shark fisheries, with Indonesia reported to be the world’s largest lander of elasmobranchs [20,25,26].

The demand for shark fin in Southeast Asia is largely driven by culturally significant dishes such as shark fin soup, a dish that is steeped in long-standing traditions and is viewed as a symbol of luxury when it is consumed [19]. Beyond culinary use, shark fin soup symbolizes affluence and prestige, particularly during significant events such as weddings and banquets [27,28]. Additionally, it is believed to promote health and offer numerous benefits to the skin, all of which contribute to its enduring popularity, consumption and cultural importance [19,27–30].

Shark fins can be purchased easily from traditional Chinese medicine (TCM) shops, wet markets in Singapore, or dried goods retailers. The species of shark that a fin came from is rarely, if ever described and when it is, whether that identification is accurate or not is questionable. Moreover, because different species of shark have been shown to accumulate toxic metals at different rates, not knowing the species of shark consumed poses risks to consumers by potentially exposing them to unsafe concentrations of toxic metals [31–33].

Visual methods of identification that rely on the morphological characteristics of the processed fin are largely ineffective in determining the species of shark that a fin came from, especially fins that are heavily processed [34–36]. However, when a fin still has diagnostic features visible (e.g. colouration or shape) preliminary species identifications can be made using a key and then confirmed by molecular techniques or an expert [36,37]. Consequently, morphological identifications alone can give incomplete and inaccurate results [38]. Thus, molecular methodologies such as DNA barcoding are employed since they operate independently of the morphological state of specimens. This method involves examining species-specific differences in DNA sequences, which are subsequently matched to known species on publicly accessible global databases [39–43]. Upon identifying the species, extracting information on trade regulations and conservation status becomes relatively straightforward. Such methods can also be applied to shipments containing processed specimens, effectively preventing the concealment of CITES-listed species as goods that are not trade-regulated [44–47]. This study seeks to further previous work performed in Singapore and aid in the monitoring of the shark fin trade to determine the impact of new, and future regulations implemented to protect and control the wildlife trade.

## 2. Methods

### 2.1. Samples collection

Between 16 and 30 January 2024, 684 shark fin samples were purchased from TCM shops (*n* = 209), wholesalers (*n* = 29), dried goods retailers (*n* = 441) and wet markets (*n* = 5) across Singapore. Retailers were selected from a curated list of 45 shops known to sell shark fins. At the time of collection this was all-known vendors in Singapore, in total, 31 vendors were visited. When possible, samples were taken from large, mixed and unspecified containers across a variety of sizes to ensure a diverse collection of shark fins. In cases where fins came from mixed bags or containers, care was taken to ensure all shark fins were individual and separate from one another. All fins were processed, dried and not possible to identify via visual methods. Fins were purchased individually and by weight, individual fins cost between USD 9 and 18 and the price per kg ranged between USD 160 and 880 per kg. Purchased shark fins were then stored at room temperature until further processing.

### 2.2. DNA extraction and PCR amplification

DNA extraction was performed following the Qiagen DNeasy Blood and Tissue Kit protocol with the following modifications: sample tissues weighed between 15 and 25 mg and the final elution volume was 50 μl. Polymerase chain reaction (PCR) was conducted in a 25 μl volume, with the following compositions: 12.5 μl GoTaq Green Master Mix, 1 μl Forward Primer at 10 μM, 1 μl Reverse Primer at 10 μM, 1 μl BSA (20 mg ml^−1^), 7.5 μl nuclease-free water and 2 μl of undiluted DNA template. Using the forward primer mlCOIintF (5′-GGW ACW GGW TGA ACW GTW TAY CCY CC−3′) [48] and reverse primer LoboR1 (5′-TAA ACY TCW GGR TGW CCR AAR AAY CA−3′) [49] we attempted to amplify an approximate 350 bp fragment of the mitochondrial cytochrome c oxidase subunit I (COI) gene. PCR thermal cycling conditions include five repeats of 94°C for 30 s, 48°C for 2 min, 72°C for 1 min, 35 repeats of 94°C for 30 s, 54°C for 2 min, 72°C for 1 min, then 72°C for 5 min [50]. Successful PCR amplification was confirmed by gel electrophoresis using a 1% TAE agarose gel.

### 2.3. Species identification

PCR products were Sanger Sequenced by Bio-Basic Asia Pacific. Quality control was performed with Geneious Prime v2024.0 [51], and only sequences that contained no ambiguous base calls and had clear, well-defined peaks were used in downstream analysis. Sequences were then queried in both the Barcode of Life Data System (BOLD) [52] and the Nucleotide BLAST (BLASTn) [53] function in Genbank to make species identifications. To be considered an accurate species identification, we used two criteria: (i) BOLD indicated a solid identification with no closely allied congeneric species known, and (ii) the same species was returned as the top match in Genbank [18].

## 3. Results

**Figure 1.**
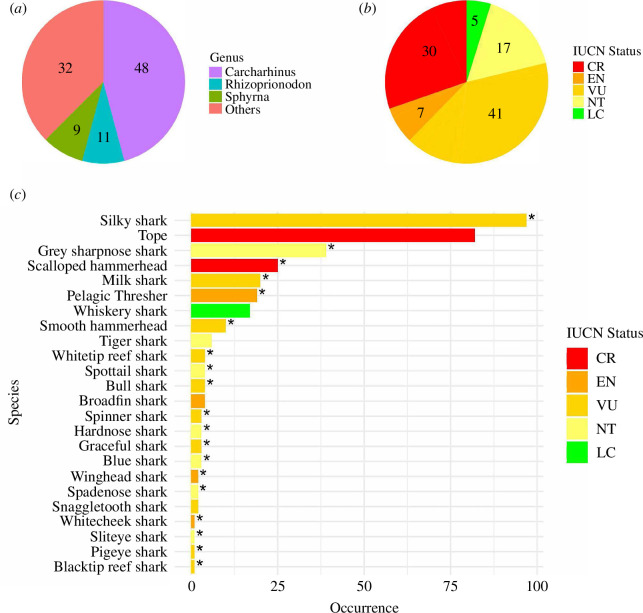
(*a*) Pie chart showing the distribution of identified samples by genus. (*b*) Pie chart showing the distribution of identified samples by IUCN status. (*c*) Bar chart showing the occurrence of samples identified at the species level. Asterisk (*) indicates species that are listed in CITES Appendix II. Numbers on pie charts indicate the per cent of each component.

After quality control, the sequence length ranged from a maximum of 272 bp to a minimum of 96 bp to a maximum of 272 bp. Out of 684 samples, 547 were successfully identified at either the genus or species level, 137 sequences failed to amplify or produce a sequence that could be used in species identifications (e.g. contained ambiguous base calls or did not have well-defined peaks). Of those that could be identified to the genus or species level, we identified 24 species spanning 16 genera, with 19 of the identified shark species currently listed on the 2024 CITES Appendices II list (*n* = 353, table 1 and figure 1). Two of the 24 species are classified as critically endangered by the IUCN (*n* = 107), with four classified as endangered (*n* = 26), ten as vulnerable (*n* = 145), and seven as near threatened (*n* = 58). One species was classified as least concern (*n* = 17) (table 1 and figure 1). Among the top five identified species, two are classified as critically endangered by the IUCN (*n* = 107), two are classified as vulnerable (*n* = 117) and one is classified as near threatened (*n* = 39). Altogether, these constituted 263 of the 547 successfully identified samples, or 48% of the total samples. The most frequently identified species was *Carcharhinus falciformis* (*n* = 97; vulnerable), followed by *Galeorhinus galeus* (*n* = 82; critically endangered) and *Rhizoprionodon oligolinx* (*n* = 39; near threatened). We were unable to identify 194 fins beyond the level of genus, the majority of these came from the genus Carcharhinus (*n* = 145), with 31 from the genus Mustelus, 12 from the genus Sphyrna, and two from each of the genera Glyphis, Hemigaleus and Rhiozoprionodon (table 1 and figure 1).

**Table 1.** Details of scientific name, common name, occurrence, relative occurrence, IUCN Red List status (CR = critically endangered, EN = endangered, VU = vulnerable, NT = near threatened, LC = least concern), and CITES list status of all identified samples.

scientific name	common name	occurrence	occurrence %	IUCN status	CITES
*Carcharhinus falciformis*	silky shark	97	18	VU	II
*Galeorhinus galeus*	tope	82	15	CR	not listed
*Rhizoprionodon oligolinx*	grey sharpnose shark	39	7	NT	II
*Sphyrna lewini*	scalloped hammerhead	25	5	CR	II
*Rhizoprionodon acutus*	milk shark	20	4	VU	II
*Alopias pelagicus*	pelagic Thresher	19	3	EN	II
*Furgaleus macki*	whiskery shark	17	3	LC	not listed
*Sphyrna zygaena*	smooth hammerhead	10	2	VU	II
*Galeocerdo cuvier*	tiger shark	6	1	NT	not listed
*Carcharhinus leucas*	bull shark	4	1	VU	II
*Carcharhinus sorrah*	spot-tail shark	4	1	NT	II
*Lamiopsis temminckii*	broadfin shark	4	1	EN	not listed
*Triaenodon obesus*	whitetip reef shark	4	1	VU	II
*Carcharhinus amblyrhynchoides*	graceful shark	3	1	VU	II
*Carcharhinus brevipinna*	spinner shark	3	1	VU	II
*Carcharhinus macloti*	hardnose shark	3	1	NT	II
*Prionace glauca*	blue shark	3	1	NT	II
*Eusphyra blochii*	winghead shark	2	<1	EN	II
*Hemipristis elongata*	snaggletooth shark	2	<1	VU	not listed
*Scoliodon laticaudus*	spadenose shark	2	<1	NT	II
*Carcharhinus amboinensis*	pigeye shark	1	<1	VU	II
*Carcharhinus dussumieri*	whitecheek shark	1	<1	EN	II
*Carcharhinus melanopterus*	blacktip reef shark	1	<1	VU	II
*Loxodon macrorhinus*	sliteye shark	1	<1	NT	II
*Carcharhinus* spp.	N/A	145	27	N/A	All CITES II
*Mustelus* spp.	N/A	31	6	N/A	none listed
*Sphyrna spp.*	N/A	12	2	N/A	All CITES II
*Glyphis spp.*	N/A	2	<1	N/A	All CITES II

## 4. Discussion

We successfully identified 353 samples to the species level, and 194 to the genus level with 137 samples failing to produce identifiable DNA sequences. It is likely that the inevitable degradation of sample DNA that occurs throughout the drying and processing of fins for sale is responsible for these failures. This DNA degradation necessitates the use of primers that amplify a fragment of reduced size, this smaller fragment lacks the resolving power of a full-length COI gene region, and when the known slow rate of evolution in sharks [54] is considered along with the short DNA fragment used, it is not surprising that some samples could only be resolved to the genus level. However, it should be noted that the vast majority of the genus-only level identifications made belong to *Carcharhinus* spp. All members of this genus are listed in CITES Appendix II meaning this information is still valuable.

The most commonly identified species was *C. falciformis* (*n* = 97, 18% occurrence), commonly known as the silky shark. It is considered vulnerable on the IUCN Red List and is listed in Appendix II of CITES. In Asia, *C. falciformis* is one of the most frequently encountered species in shops and trading ports [46,55–57]; this is despite the implementation of regulations to monitor, control and prevent trade that is incompatible with its continued existence [25,46,55]. Previous studies frequently identify *C. falciformis* as a commonly encountered species in trade regions such as Singapore, Hong Kong and Indonesia [40,56,57]. Consequently, it has been suggested that there is an urgent need to re-evaluate the CITES status of this species and include it in Appendix I of CITES [58]; the frequent occurrence of *C. falciformis* in this work adds weight to this claim. This suggested re-evaluation is primarily due to reports of rapid declines in its populations throughout the Pacific, Indian and Atlantic Oceans [3,59,60]. Additionally, due to life history characteristics (e.g. a long duration to sexual maturity), this species is particularly vulnerable to overfishing and may not have the capacity to reproduce fast enough to offset population losses incurred through overexploitation [6].

The second most frequently identified species (*n* = 82, 15% occurrence) was *G. galeus*, also known as the tope shark or school shark. While not listed by CITES, this species is considered critically endangered by the IUCN. Population genetic studies have shown that *G. galeus* is generally restricted to coastal areas where largely distinct populations can be observed [61]. Further reinforcing this population structure, the migratory range of *G. galeus* is generally restricted within ocean basins where migrations are primarily driven by temperature fluctuations or reproductive needs [62,63]. Further work employing advances in DNA sequencing and sequence assembly (e.g. whole genome sequencing, or reduced representation libraries) may allow us to assign individual fins of this species back to specific stocks or populations, and in doing so could allow the development of specific conservation strategies ensuring that fishing of *G. galeus* only occurs in sustainably managed fisheries rather than fisheries that are known to be threatened or fished unsustainably.

The third most commonly observed species was *R. oligolinx* (*n* = 39, 7% occurrence), or the grey sharp-nosed shark. *R. oligolinx* is considered near threatened by the IUCN and is listed on CITES Appendix II. It is often caught by gillnet fleets in the Java Sea where it is the main target of fisheries in the region [64]. While utilization of the species for human consumption in the Java Sea is currently considered sustainable, populations in other parts of the world are likely fished unsustainably. For example, research performed in the waters of Mumbai, India shows that most *R. oligolinx* caught in the region are not sexually mature [65], and increasing demand for this species has contributed to a rise in annual catches since 2012, with research showing that harvest of this species needs to be reduced by 40% to ensure sustainability [65]. Without the enforcement of appropriate fishing and trade regulations, it is likely this pressure will continue to increase with negative consequences for *R. oligolinx* populations.

Species with cosmopolitan distributions that span multiple national exclusive economic zones (EEZs) are recognized as having a higher incidence in the shark fin trade [7], these distributions make stock management and conservation efforts more difficult owing to their wide-ranging habitats and increased exposure to international fishing pressures [55,66]. In contrast, non-cosmopolitan species that are regionally restricted, such as the *R. oligolinx*, are subject to more stringent management guidelines tied to their locality [55,64]. Understanding the importance of multilateral efforts, technological solutions and stronger enforcement creates very different outcomes in the conservation and protection of CITES-listed endangered species (e.g. sharks) [67]. Although CITES and IUCN provide one such framework for international fisheries regulations, regional and species-specific multilateral agreements could further improve protection and conservation measures in multi-territory fisheries.

Similar to this work, Drescher *et al*. [40] and Shen *et al*. [68] also identified *C. falciformis*, *G. galeus* and *R. oligolinx* as the most frequent species being traded in Singapore. However, the current study had a lower occurrence of Hammerhead sharks, *Sphyrna* spp. 9%, in comparison to previous surveys where it accounted for 12%–15% of the samples identified [40,68]. There are several possible explanations for the lower occurrence of this species in our study; (i) it is possible that the addition of *Sphyrna* spp. to CITES Appendix II in February 2023 enhanced its management and contributed to a relatively lower occurrence in this work, (ii) the market could be cyclical meaning the species caught varies with global or regional fishing trends, (iii) our sampling is not reflective of the true diversity of species found in Singapore when collections were made, (iv) alternatively, it could be reflective of an overall population decline in *Sphyrna* spp. It is thus important to continue monitoring the relative abundance of hammerhead and other newly regulated species in the shark fin trade to determine whether newly implemented policies and regulations are working and having the desired effect.

A more comprehensive, better implemented and enforced labelling of regulated wildlife such as sharks can help mitigate illegal and unregulated trade and prevent biodiversity loss. During the collection of shark fins for this research, it was evident that the species of shark that a fin came from was not indicated. Instead, labels used generic names or terms (e.g. ‘shark fin’ or the common Mandarin name ‘鱼翅”, yu chi.) and most were stored in clear boxes or arranged according to size, but species, source or information detailing when the product was caught is not available. The long shelf life of dried fins and the rate of product turnover in each shop mean it is extremely difficult to determine exactly when the shark that a fin belonged to was caught. Consequently, the possibility that these sharks were caught or traded before current trade regulations were implemented cannot be eliminated. Ultimately, ambiguous labelling of these products impedes regulatory actions meant to protect vulnerable species. Ideally, traded fins should be accompanied by information on the species’ name, along with the date and area of capture.

From a human health perspective, inadequate labelling can also lead to the unintentional consumption of shark products that pose a higher risk of toxic metal exposure in consumers [32]. Further amplifying the need for accurate and comprehensive labelling of species, toxic metals, including mercury, arsenic, cadmium and lead do accumulate at different rates and concentrations dependent upon the species and its habitat [32,33]. Given these differences, accurate and comprehensive labelling of fins is needed for well-informed consumer choices and the avoidance of species that are acknowledged to contain significantly higher concentrations of toxic metals.

While this study only offers insights into shark products sold during a two-week period in January 2024 when samples were collected, it highlights the prevalence and accessibility of the trade of regulated and endangered species for sale in Singapore. It also emphasizes the need to consistently monitor the species of shark fins being traded both in the region and globally, doing this will better inform international policies surrounding shark fishing and trade. Future work should include analysis of samples collected at regular intervals throughout the year to shed light on seasonal variations in the sale of shark products and offer a better understanding of the shark fin market as a whole. Continued, structured monitoring and accurate species identifications are crucial to evaluating the effectiveness of fishing and trade regulations designed to prevent unsustainable shark fishing.

## Data Availability

Data used are available in the electronic supplementary material [69].
